# MicroRNA expression profiles of stress susceptibility and resilience in the prelimbic and infralimbic cortex of rats after single prolonged stress

**DOI:** 10.3389/fpsyt.2023.1247714

**Published:** 2023-08-24

**Authors:** Gengdi Huang, Javed Iqbal, Dan Shen, Yan-xue Xue, Mei Yang, Xiaojian Jia

**Affiliations:** ^1^State Key Laboratory of Chemical Oncogenomics, Guangdong Provincial Key Laboratory of Chemical Genomics, Peking University Shenzhen Graduate School, Shenzhen, China; ^2^Department of Addiction Medicine, Shenzhen Engineering Research Center for Precision Psychiatric Technology, Shenzhen Clinical Research Center for Mental Disorders, Shenzhen Kangning Hospital, Shenzhen Mental Health Center, Shenzhen, China; ^3^Henan Key Laboratory of Medical Tissue Regeneration, Xinxiang Medical University, Xinxiang, China; ^4^National Institute on Drug Dependence and Beijing Key Laboratory of Drug Dependence, Peking University, Beijing, China; ^5^Affiliated Mental Health Center, Southern University of Science and Technology, Shenzhen, China; ^6^Clinical College of Mental Health, ShenZhen University Health Science Center, Shenzhen, China; ^7^School of Mental Health, Jining Medical University, Jining, China; ^8^School of Mental Health, Anhui Medical University, Hefei, China

**Keywords:** transcriptome, stress, susceptibility, resilience, prefrontal cortex

## Abstract

The experience of traumatic stress can engender lasting memories associated with the trauma, often resulting in post-traumatic stress disorder (PTSD). However, only a minority of individuals develop PTSD symptoms upon exposure. The neurobiological mechanisms underlying the pathology of PTSD are poorly understood. Utilizing a rat model of PTSD, the Single Prolonged Stress (SPS) paradigm, we were able to differentiate between resilient and susceptible individuals. Fourteen days after the SPS exposure, we conducted the behavioral analyses using Elevated Plus Maze (EPM) and Open Field (OF) tests to identify male rats as trauma resilient or susceptible. We focused on the microRNA (miRNA) profiles of the infralimbic (IL) and prelimbic (PL) cortical regions, known to be crucial in regulating the stress response. Our investigation of stressed rats exposed to the SPS procedure yielded divergent response, and differential expression microRNAs (DEmiRs) analysis indicated significant differences in the IL and PL transcriptional response. In the IL cortex, the GO analysis revealed enriched GO terms in the resilient versus control comparison, specifically related to mitogen-activated protein kinase and MAP kinase signaling pathways for their molecular functions as well as cytosol and nucleoplasm for the biological process. In the susceptible versus resilient comparison, the changes in molecular functions were only manifested in the functions of regulation of transcription involved in the G1/S transition of the mitotic cell cycle and skeletal muscle satellite cell activation. However, no enriched GO terms were found in the susceptible versus control comparison. In the PL cortex, results indicated that the DEmiRs were enriched exclusively in the cellular component level of the endoplasmic reticulum lumen in the comparison between resilient and control rats. Overall, our study utilized an animal model of PTSD to investigate the potential correlation between stress-induced behavioral dysfunction and variations in miRNA expression. The aforementioned discoveries have the potential to pave the way for novel therapeutic approaches for PTSD, which could involve the targeted regulation of transcriptome expression.

## Introduction

The occurrence of a life-threatening event can give rise to the development of PTSD which is distinguished by intrusive memories, avoidance of stimuli that trigger recollections of the event, recurring nightmares, and persistent hyperarousal (1). The symptoms of PTSD may not become apparent immediately after the trauma and may exhibit a delayed onset. Some symptoms may show partial remission and relapse, while others may persist and worsen over time. Despite most people undergo at least one traumatic experience in their lives, only a small fraction, constituting 10–20% of the population, develop enduring manifestations of PTSD (2). Conversely, some other individuals display resilience and do not develop lasting psychological consequences (3). Resilience, which refers to the ability to adapt effectively in the face of significant adversity, is often the prevailing response pattern observed in individuals who have experienced trauma (4, 5). Understanding the neural mechanisms that differentiate individuals from those vulnerable to stress-related disorders is crucial in the prevention of psychiatric disorders and the development of innovative treatment approaches.

Studies conducted on both humans and animals have suggested that the abnormalities in neurocircuits that are linked to emotions and fear, specifically the amygdala, prefrontal cortex (PFC), and hippocampus, may contribute to the development and maintenance of symptoms of PTSD (6). Moreover, aberrations in the medial prefrontal cortex (mPFC) function have been implicated in the perpetuation of fearfulness in anxiety disorders, such as PTSD (7–9). Advanced high-precision neuroimaging and optogenetic methods have confirmed that PFC is an essential brain structure in the regulation of PTSD (10). Studies of rodents have shown that SPS leads to decreased neuronal activation in the IL, which could be a factor in the associated fear extinction deficits (11–13). In contrast, optogenetic excitation of the mPFC has been linked to increased stress resilience, and successful stress coping is associated with heightened mPFC activation following social defeat stress (14). Overall, it becomes evident that the PFC plays a pivotal role in regulating an individual’s susceptibility or resilience to stress. In light of the aforementioned considerations, more analysis to the PFC subregions, including the IL and PL cortex (15), should be further investigated.

The factors that contribute to the development of PTSD in some individuals following exposure to traumatic events remain largely unknown. Nevertheless, it is likely that alterations in the molecular mechanisms responsible for regulating stress vulnerability and adaptability are implicated in this process (16). Epigenetic modifications are primarily regulated through DNA methylation, histone modifications and the control of non-coding RNAs, and any disruption of these modifications can be detrimental to normal neuronal development and functioning, resulting in severe neuropsychiatric disorders (17, 18). Specifically, MicroRNAs (miRNAs), a class of small non-coding RNAs, have emerged as potential biomarkers for various diseases, including psychiatric disorders (19, 20). Acting as essential regulators of gene expression, miRNA primarily repress the expression of their target mRNAs (21, 22). Numerous studies have suggested that miRNAs are involved in the molecular mechanisms of fear memory (23), and stress reactions associated with PTSD (24, 25). In animal studies, altered miRNA expression in the PFC (26) and amygdala (27) of rodents exposed to traumatic stress, exhibited PTSD-like symptoms. Moreover, the circulating miRNAs were examined as potential diagnostic biomarkers in individuals with PTSD and corresponding controls (28, 29). These results indicate that the aberrant miRNA expression observed in PTSD may be correlated with immune responses and neuronal activation. The examination of alterations in gene expression in the key regions of PTSD, particular subregions of the PFC, will provide insights into the fundamental neurobiological processes, ultimately leading to individualized treatment that prevent susceptibility or promote resilience to stress. In doing so, we utilized a transcriptome analysis of differential miRNA expression with subsequent gene ontology-based pathway analysis. Our primary objective is to investigate the transcriptome associated with susceptibility and resilience phenotypes in IL and PL cortex, aiming to comprehend the underlying molecular mechanisms and identify innovative therapeutic targets and biomarkers.

## Methods

### Animals

Male Sprague–Dawley (SD) rats, approximately 3 months old, were procured from Beijing Vital River Laboratory Animal Technology Co., Ltd. These animals were housed in a controlled environment with a 12 h light/dark cycle and provided with *ad libitum* access to food and water. They were housed in groups of four per cage for 1 week prior to the commencement of the experiment. The light/dark cycle was maintained at 12 h each. Subsequently, some of the rats were randomly subjected to the SPS protocol to induce PTSD-like behavior, while the remaining rats were kept as controls in separate cages. The behavioral experiments were conducted during the dark phase of the cycle. All animal-related experiments were conducted in accordance with the National Institutes of Health Guide for the Care and Use of Laboratory Animals and the Biomedical Ethics Committee of Peking University’s regulations on animal use and protection. The Biomedical Ethics Committee of Peking University also approved the protocol for animal use and protection.

### Experimental design

**Figure 1:**
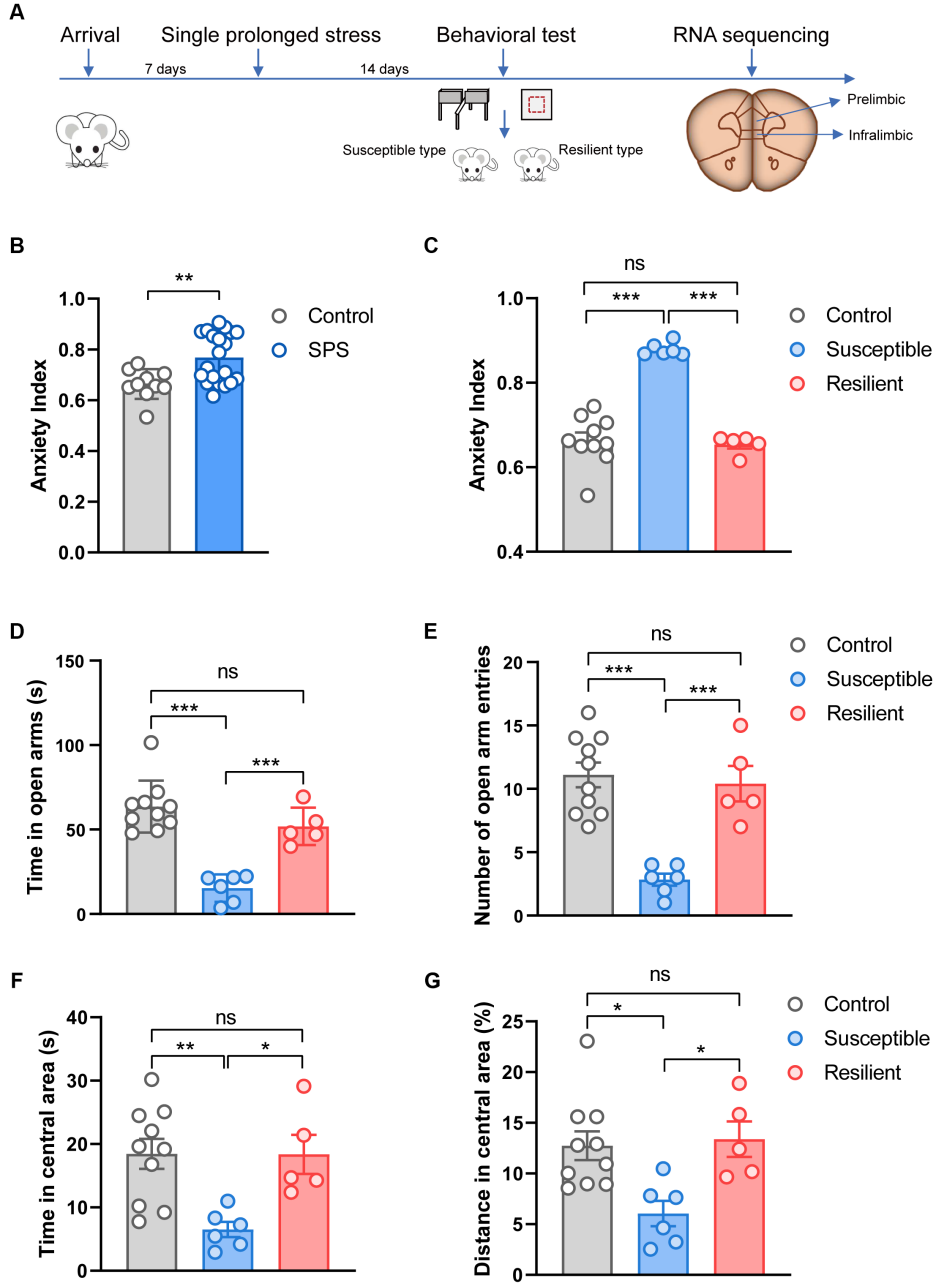
Experimental design and behavioral test. **(A)** Overview of experimental design. **(B)** Effect of SPS on anxiety index in control and SPS group. **(C)** Effect of SPS on anxiety index in control, susceptible, and resilient group. **(D)** Time spent in open arms. **(E)** Number of open arm entries. **(F)** Time spent in central area. **(G)** Distance spent in central area.

The experimental design is shown in Figure 1A. Thirty rats (260–280 g) were acclimatized for 7 days, then twenty underwent the SPS stressors, and the other ten rats as control. After undisturbed for 14 days, they were tested on OF and EPM. The animals were sacrificed after the behavioral test, the PL and IL cortex were dissected and stored at −80°C until used for RNA sequencing analysis.

### Single prolonged stress

An animal model for PTSD-like symptoms was constructed by the SPS procedure as previously described (30). The SPS rat model is widely regarded as one of the most reliable models for studying PTSD. The procedure included a 2 h restraint stress in a plastic restrainer, followed by a 20 min forced swim at a depth of 40 cm and a temperature of 25°C. After a 15 min rest, the rats were anesthetized using diethyl to induce unconsciousness.

### Behavioral test

The OF and EPM test was based on our previous studies (30) and was conducted in a procedure room under dim light conditions. To eradicate the previous animal’s odor, the apparatus underwent a cleaning procedure utilizing a towel drenched in a 75% ethanol solution. These two tests were recorded for 5 min per rat and then analyzed with LabMaze v3.0 (Zhongshi Technology, China). In the OF test, the total travel distance and the time spent in the central area were counted to measure the rats’ behavior. In the EPM test, the number of open/closed arm entries and the time spent in the open/closed arms were recorded. Vulnerable and resilient subpopulations of rats emerge after SPS. According to the previous study (31), individual animals were retrospectively classified as having behavioral responses according to the anxiety index (AI), which is calculated as 1 − [(time spent in open arm/total time on the maze)/2 + (number of entries to the open arms/total exploration on the maze)/2]. For classification criteria, one standard deviation below the mean of the control group’s anxiety index as resilient individuals, and one standard deviation above the mean of the the control group’s anxiety index as susceptible individuals. After SPS procedure, 6 rats reached susceptible criteria and 5 rats reached resilient criteria. A subset of 12 animals were selected for RNA sequencing based on the anxiety index: 4 out of 5 resilient individuals, 4 of 6 susceptible individuals, and 4 out of 10 control animals were randomly chosen.

### RNA sequencing and analysis

The animals were sacrificed after the EPM and OF tests, and samples of IL and PL cortex were dissected from each animal. Then, the tissue samples were sent to BGI Co., LTD (Shenzhen, China), and the BGISEQ-500 platform was applied to perform RNA sequencing. DEmiRs were determined based on |log2(fold change) | ≥ 0.5 and *q* value (Adjusted *p*-value) < = 0.05. To gain insight into the biological roles of the differentially expressed miRNAs, we used the gene ontology (GO)-term and the Kyoto Encyclopedia of Genes and Genomes (KEGG) pathway analyses. All analysis of RNA sequencing data was performed by DR. TOM online system provided by BGI.^1^

### Statistical analysis

The behavioral data were expressed as mean ± SEM. The data were analyzed using an analysis of *t*-test or one-way ANOVA followed by Bonferroni’s multiple comparison test (see Results). Fisher’s exact test was used to compare the distribution of each GO classification or KEGG pathway in the target miRNA set, and the *q* value (Adjusted *p*-value) < = 0.05. Statistical significance was set at *p* < 0.05. *, **, and *** depicting *p* < 0.05, *p* < 0.01, and *p* < 0.001, respectively.

## Results

### Divergent responses on EPM and OF after SPS

Fourteen days after the SPS procedure, animals were tested on OF and EPM. Results analysis showed that the anxiety index in the SPS group was significantly higher compared to the control group (*t* = 3.10, *p* < 0.001, Figure 1B). To better understand the susceptibility and resilience to stress, SPS animals were classified into two groups, namely susceptible and resilient, based on their performance in the anxiety index assessment. These groups differed significantly on anxiety index (F_2, 18_ = 54.25, *p* < 0.001, Figure 1C), specifically, the susceptible group exhibited a statistically significant difference in anxiety index as compared to both the resilient and control groups (*p* < 0.001). However, no significant difference was observed between the resilient and control groups. In the EPM test, these groups differed significantly on time (F_2, 18_ = 27.19, *p* < 0.001, Figure 1D) and the number of entries (F_2, 18_ = 18.84, *p* < 0.001, Figure 1E) into the open arms, but the significant difference only appear in susceptible group when compare to control and resilient group (*p* < 0.001). In the OF test, the susceptible group displayed more anxiety behaviors, and tended to spend less time (F_2, 18_ = 7.421, *p* < 0.001, Figure 1F) and trace on central area (F_2, 18_ = 6.414, *p* < 0.001, Figure 1G), in contrast, no significant changes were observed in resilient group and control group.

### Regional-specific changes in miRNAs expression

**Figure 2:**
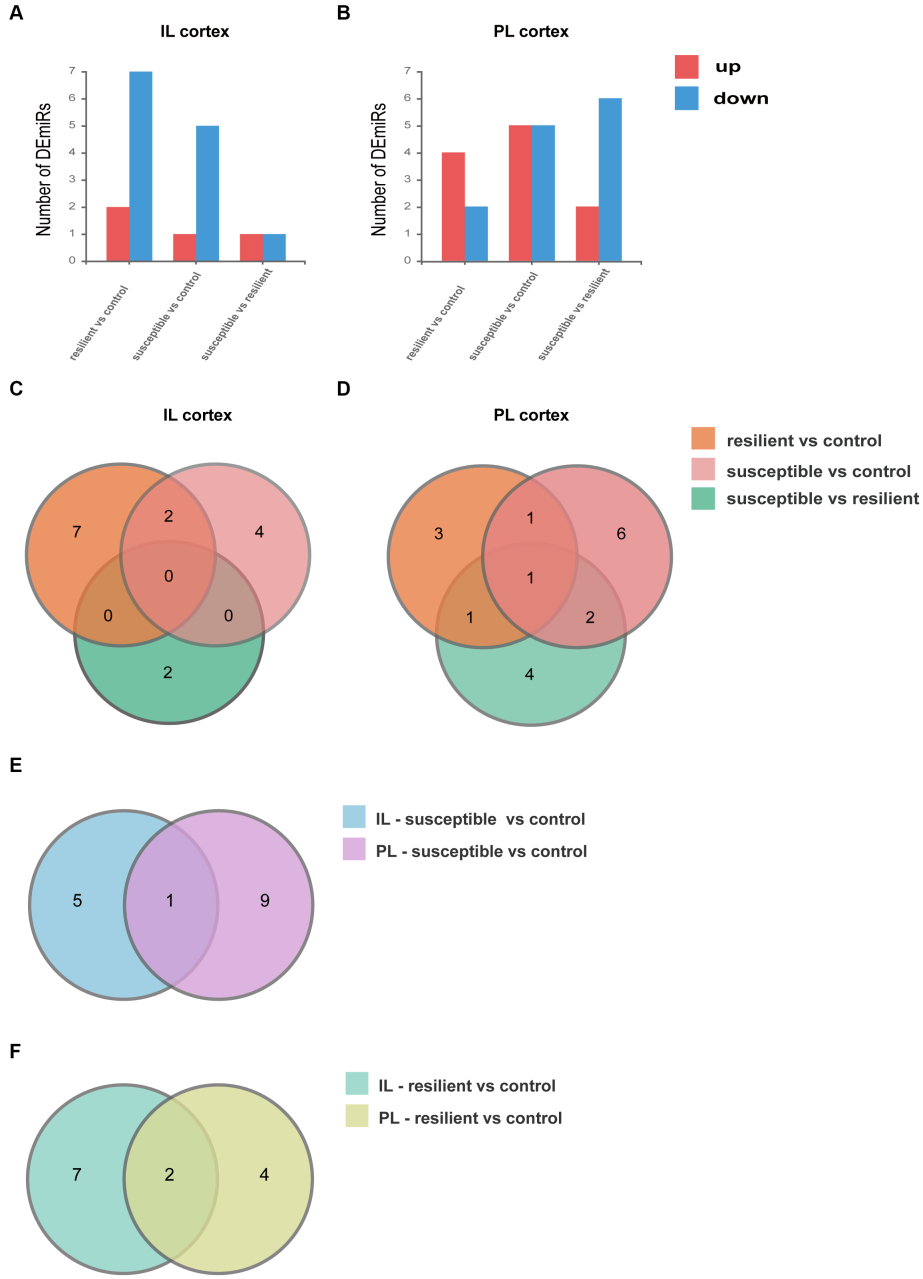
Differentially expressed microRNA (DEmiRs). **(A)** DEmiRs within the IL cortex. **(B)** DEmiRs within the PL cortex. **(C)** Venn diagram of DEmiRs in IL cortex. **(D)** Venn diagram of DEmiRs in PL cortex. **(E)** Venn diagram of susceptible versus control comparison between IL and PL cortex. **(F)** Venn diagram of resilient versus control comparison between IL and PL cortex.

We performed small RNA-seq on the IL and PL cortex and compared the miRNA expression between groups. Differential expression analysis of each subset reveals significant differences in both genes being up- and downregulated in IL and PL cortex. For DEmiRs in the IL cortex, we identified 9 DEmiRs in the resilient versus control comparison, 6 DEmiRs in the susceptible versus control comparison, and 2 DEmiRs in resilient versus susceptible comparison (Figure 2A). For DEmiRs in PL cortex, there are 6 DEmiRs in the resilient versus control comparison, 10 DEmiRs in the susceptible versus control comparison, and 8 DEmiRs in resilient versus susceptible comparison (Figure 2B). Venn diagram demonstrating shared DEmiRs were plotted, comparison of resilient/control and susceptible/control profiles showed only 2 common DEmiRs in IL cortex (Figure 2C), and no common DEmiRs in PL cortex. However, between resilient/control and resilient/susceptible, we only found 1 overlapping DEmiR, and we also found 2 overlapping DEmiRs between resilient/control and resilient/susceptible in PL cortex (Figure 2D). Only one gene overlapped between IL and PL cortex in susceptible versus control comparison (Figure 2E), and two genes overlapped between IL and PL cortex in resilient versus control comparison (Figure 2F).

### Gene ontology analysis of differentially expressed miRNAs

**Figure 3:**
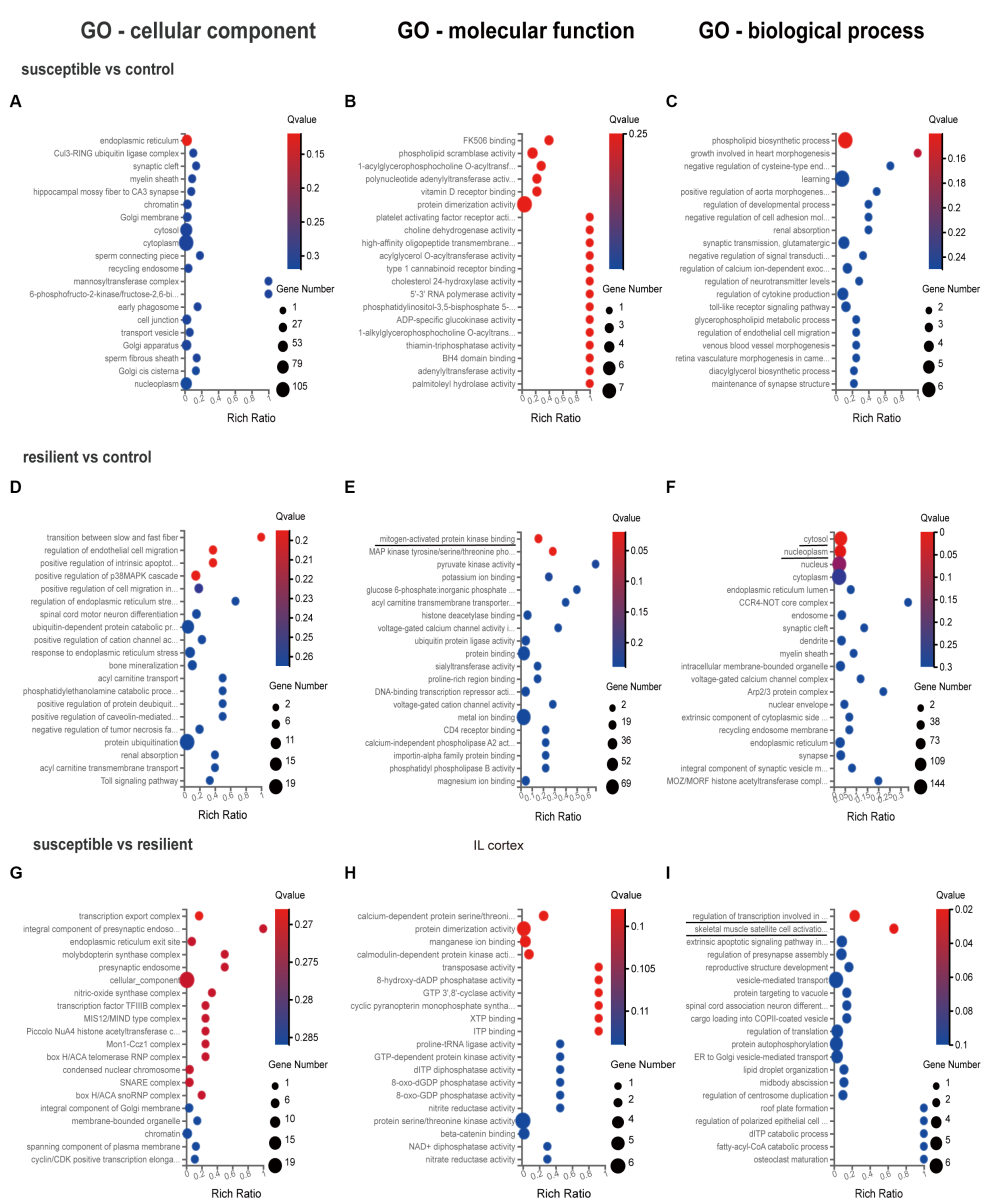
Gene Ontology enrichment analysis for DEmiR in IL cortex. **(A–C)** GO terms in susceptible versus control comparison. **(D–F)** GO terms in resilient versus control comparison. **(G–I)** GO terms in susceptible versus resilient comparison.

**Figure 4:**
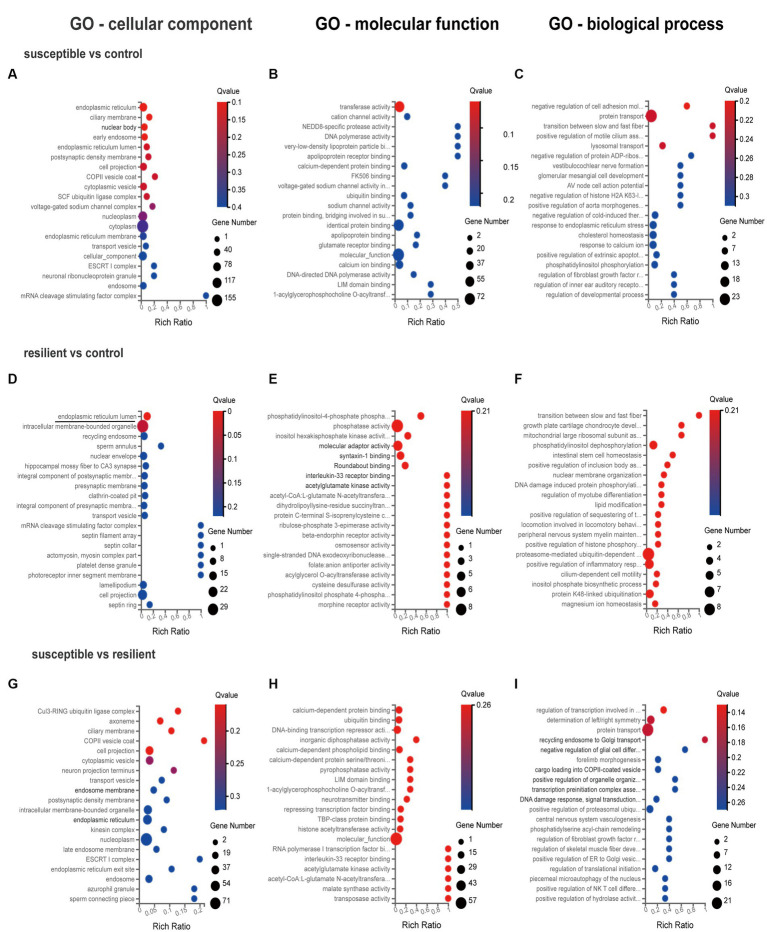
Gene Ontology enrichment analysis for DEmiR in PL cortex. **(A–C)** GO terms in susceptible versus control comparison. **(D–F)** GO terms in resilient versus control comparison. **(G–I)** GO terms in susceptible versus resilient comparison.

GO enrichment analysis was used for gene annotation and function description, containing three sub-ontologies of defined terms that describe gene product attributes, such as biological processes (BP), cellular components (CC), and molecular functions (MF). In IL cortex, the gene enrichment of miRNA and coexpressed mRNAs in susceptible versus control comparison did not significantly participate in any signaling pathways (Figures 3A–C). GO analysis of resilient versus control comparison showed that changes in the cellular and molecular function were enriched in the mitogen-activated protein kinase (*q* value = 0.04) and MAP kinase signaling pathways (*q* value = 0.04) (Figures 3D,E). And the changes in the biological process of DEmiRs were enriched in the cytosol (*q* value<0.01) and nucleoplasm (*q* value = 0.01) (Figure 3F). While in susceptible versus resilient comparison, the changes in biological functions except cellular and molecular were only manifested in the functions of regulation of transcription involved in G1/S transition of mitotic cell (*q* value = 0.02) and skeletal muscle satellite cell activation (*q* value = 0.02) (Figures 3G–I). In susceptible vs control, no important cellular, molecular and biological functions were found in PL cortex (Figures 4A–C). There were no important GO enrichments at molecular and biological levels (Figures 4E,F). Similarly, the GO enrichments were not significant at cellular, molecular and biological levels in susceptible vs resilient comparison (Figures 4G,H).

### KEEG ontology analysis of differentially expressed miRNAs

**Figure 5:**
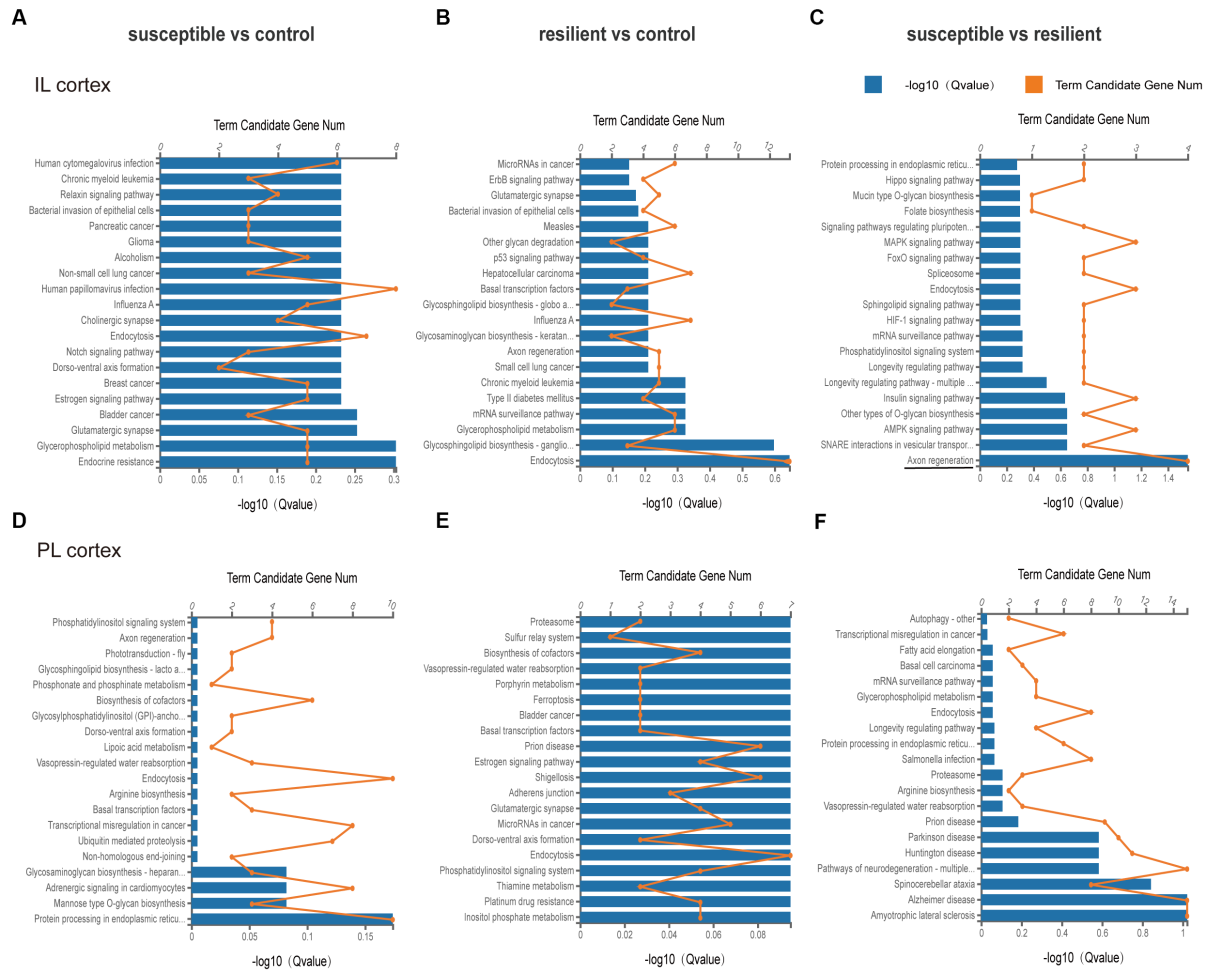
KEEG pathway analysis of DEmiRs in different comparisons. **(A–C)** KEGG pathway enrichment analysis of DEmiRs in IL cortex was performed in susceptible versus control, resilient versus control, and susceptible versus resilient comparisons. **(D–F)** KEGG pathway enrichment analysis of DEmiRs in PL cortex was performed in susceptible versus control, resilient versus control, and susceptible versus resilient comparisons.

Based on KEEG classification, KEGG analysis in the IL cortex mainly involved Axon regeneration in susceptible versus resilient comparison (*q* value = 0.028, Figure 5C). However, no altered pathways were obtained in susceptible versus control and resilient versus control comparisons (Figures 5A, B). As for PL cortex, KEGG analysis suggested that DEmiRs did not involve any pathway between group comparison (Figures 5D–F).

Overall, our research employed an animal model of PTSD to explore the association between stress-induced behavioral issues and alterations in miRNA expression. Results analysis of rats exposed to the SPS procedure revealed disparate reactions, and DEmiRs analysis revealed considerable differences in the IL and PL transcriptional response.

## Discussion

In this study, the SPS procedure was utilized to induce different stress response phenotypes, as determined by anxiety index. We profiled the miRNAs in the IL and PL cortex of resilient and susceptible rats and subsequently explored the biological alterations of the DEmiRs by enrichment analysis.

SPS-exposed rats exhibited more severe anxiety behaviors than control rats after a long duration. We also found divergent responses among the SPS-exposed rats, resulting in two distinct phenotypes: susceptible and resilient. Animal models of PTSD have proven to be highly effective in assessing the underlying biochemical mechanisms that contribute to the development of long-lasting impairments. These models also facilitate the identification of biomarkers and the testing of potential therapeutic interventions (32, 33). The behavioral impairments observed in SPS rats resemble the key symptoms associated with PTSD, indicating the reliability of the model (34–36). Moreover, previous studies also showed divergent responses to the SPS in rats (31, 37, 38), indicating the ability of SPS to induce behavioral alterations that reproduce PTSD characteristics only in a subset of exposed individuals. According to the anxiety index, approximately 30% of the SPS-exposed rats can be considered as susceptible, while other 25% of rats as resilient. The current findings further support the reliability of the SPS as an animal model for PTSD, as it mimics the heterogeneous response observed in individuals with PTSD. Individuals who are prone to stress often encounter difficulties in adjusting to stressors and may manifest maladaptive reactions over time. On the other hand, resilient individuals possess the ability to perceive adversity as relatively harmless and can establish adaptive physiological and psychological coping strategies (39). Each individual may perceive exposure to stressful events differently, and the resulting effects can be long-lasting, contingent upon the degree of stress resilience or vulnerability of each individual. Randomly assigning rats to different groups could reduce individual differences, and detecting anxiety levels prior to the SPS procedure would help to identify rats prone to stress. In animal research, individual behavioral profiling allows investigating neurobiological alterations related to susceptibility or resilience in animal models of PTSD and is utilized here to examine the activation of differential transcriptome expression of the stress system associated with trauma susceptibility or resilience.

Our findings demonstrated that the IL and PL cortex have different miRNA expression patterns, suggesting differential contributions within subregions to the stress response. Interestingly, transcriptional regulation of each subregion may have an influential, expression-independent role in PTSD-like behavior, even though only a few modified transcripts are differentially expressed. The mPFC is known to be involved in behavioral and executive functions, which are impacted by stress in both rodents and humans and are disrupted in individuals with stress-related disorders like PTSD and Major Depressive Disorder (40, 41). Human fMRI studies have revealed that increased prefrontal activity may serve as a resilience factor against PTSD, indicating that improved top-down control of subcortical regions promotes more effective emotion regulation (42). Thus, investigating the mPFC, particularly when its impact on vulnerability to stress, is crucial for the discovery of novel biomarkers that can distinguish between resilience and vulnerability to stress, offering valuable insights into the prevention and treatment of stress-related psychiatric disorders. In the present study, we proposed that the subregions of the mPFC, specifically the PL and IL cortex, would be impacted differently by traumatic stress after exposing rats to the SPS procedure. It has been observed that SPS causes a hypoactivity of the ventromedial prefrontal cortex (vmPFC) and hyperactivity of the amygdala, leading to a lack of top-down control, which is thought to be the source of the abnormal fear responses observed in SPS, and is similarly seen in PTSD patients (12, 43). Evidence also suggests that SPS-induced extinction impairments can be reversed with chemogenetic activation of the IL, demonstrating the crucial role of IL cortex in stress-related behavioral deficits (44). Also, the IL is a vital component in the processes of extinction, extinction recall, and reinstatement of conditioned fear (45), and damage or inactivation of this structure leads to extinction memory deficits that are similar to those observed in individuals with PTSD (46). Moreover, the IL region has direct and indirect projections to the intercalated neurons which suppress activation of the CeA, while the PL region sends signals to the BLA to arouse the CeA (47), which suggests differential contributions of different subregions of the mPFC. Notably, previous findings demonstrated enhancement of extinction of fear memory by chemogenetic activation of mPFC excitatory neurons only appeared in IL but not the PL region (44). Moreover, SPS was found to decrease neural activity in the IL but not PL, as observed in manganese-enhanced magnetic resonance imaging (12). Further investigation is needed to elucidate the roles of subregions in PTSD development. Moreover, gender is a fundamental factor to consider when examining PTSD, as women are two times more likely to suffer from it than men (48), consequently, more research is required in the future to explore the gender factor in PTSD.

The present study identified core PFC transcriptional signatures that are associated with behavioral phenotypes, including resilience and susceptibility. Our analysis of DEmiRs data suggests significant distinctions exist between the SPS-exposed groups in IL and PL cortex. Specially, we identified 17 DEmiRs in IL cortex and 24 DEmiRs in PL cortex. Interestingly, there was limited overlap between the IL and PL cortex comparisons, with only one gene (miR-205) overlapping between IL and PL cortex in susceptible versus control comparison, and two genes (miR-133c, miR-3,587) overlapping between IL and PL cortex in resilient versus control comparison. Previous studies indicated that miR-142, miR-124, and miR-153 regulation in the hippocampus may be important therapeutic targets for PTSD and could alleviate SPS-exposed PTSD-like behaviors (24, 25, 49). However, there is currently limited literature on PFC transcriptome changes after SPS. In the IL cortex, the GO analysis revealed enriched GO terms in the resilient versus control, including mitogen-activated protein kinase, MAP kinase signaling pathways for their molecular functions, as well as cytosol and nucleoplasm for the biological process. In the susceptible versus resilient comparison, the changes in molecular functions were primarily related to the functions of regulation of transcription involved in G1/S transition of mitotic cell cycle and skeletal muscle satellite cell activation. Notably, no enriched GO terms were found in susceptible versus control comparison. In the PL cortex, the GO enrichment analysis conducted on the target genes indicated that the DEmiRs were enriched exclusively in the cellular component level of endoplasmic reticulum lumen in the comparison between resilient and control rats. As far as we know, this study is the first to investigate the transcriptome expression profile in the mPFC after SPS in rats. A previous study examining the immediate changes to the transcriptional architecture in the PFC in response to footshock stress in rats showed no substantial changes at the single gene level immediately after the stress session. However, gene set enrichment analysis demonstrated modifications in neuronal pathways linked to glia maturation, glia–neuron communication, and synaptic activity (50). Other brain areas, including nucleus accumbens (NAc) and locus coeruleus (LC), have been investigated in rats using the SPS model, revealing the DEGs in the LC were found to be associated with morphological changes, including the regulation of actin cytoskeleton, regulation of cell size, growth factor activity, brain development and memory, and few DEGs appeared in NAc (31). However, this study only highlights different transcriptional profiles within the LC and NAc between susceptible and resilient groups, without control group.

## Conclusion

Overall, we observed that only a subset of rats exposed to a traumatic event exhibited symptoms similar to those of PTSD. Our finding has facilitated the identification of alterations in the transcriptome of the mPFC, providing novel insights into the underlying mechanisms that may account for the various phenotypic effects induced by stress. The investigation of gene expression alterations in the IL and PL and the pathway that underlie these changes will enhance our understanding of the core neurobiological processes of PTSD. Although multiple gene mutations likely contribute to PTSD in different individuals, there may be converging neurobiological gene expression changes that contribute to the divergent symptoms of PTSD.

## Data availability statement

The data presented in this study are deposited in the NCBI SRA database with accession number PRJNA1001887 and are accessible at the following link: https://www.ncbi.nlm.nih.gov/sra/PRJNA1001887.

## Ethics statement

The animal study was approved by the Biomedical Ethics Committee of Peking University. The study was conducted in accordance with the local legislation and institutional requirements.

## Author contributions

XJ, MY, and YX were instrumental in the conception and design of the work. GH, JI, and DS were involved in the acquisition of the data. GH and JI were responsible for analyzing and interpreting the data, as well as writing the article. In the course of a rigorous review process, XJ, MY, and YX have collaborated to scrutinize the article and have committed to being answerable for all elements of the project. All authors contributed to the article and approved the submitted version.

## Funding

This work was supported by grants from the Science and Technology Planning Project of Shenzhen Municipality (KCXFZ20211020164543007, 20210617155253001), the Guangdong Basic and Applied Basic Research Foundation (2021A1515012141), the Shenzhen Fund for Guangdong Provincial High-level Clinical Key Specialties (SZGSP013), the Fund of Development and Reform Commission of Shenzhen Municipality (XMHT20220104028), and the National Natural Science Foundation of China (82071498, 82101492 and 32250410298).

## Conflict of interest

The authors declare that the research was conducted in the absence of any commercial or financial relationships that could be construed as a potential conflict of interest.

## Publisher’s note

All claims expressed in this article are solely those of the authors and do not necessarily represent those of their affiliated organizations, or those of the publisher, the editors and the reviewers. Any product that may be evaluated in this article, or claim that may be made by its manufacturer, is not guaranteed or endorsed by the publisher.

## Footnotes

^1^https://biosys.bgi.com
